# Virus-like Particles from *Wolbachia*-Infected Cells May Include a Gene Transfer Agent

**DOI:** 10.3390/insects14060516

**Published:** 2023-06-02

**Authors:** Ann M. Fallon, Elissa M. Carroll

**Affiliations:** Department of Entomology, University of Minnesota, 1980 Folwell Ave., St. Paul, MN 55108, USA

**Keywords:** alpha-proteobacteria, bacteriophage, GTA, mosquito cell culture, GH_25 hydrolase, +1 ribosomal frameshift, horizontal gene transfer

## Abstract

**Simple Summary:**

*Wolbachia* are obligate intracellular bacteria that occur in insects and filarial worms. Strains that infect insects have genomes that encode mobile genetic elements that are typically lost as obligate intracellular bacteria undergo genomic streamlining. These mobile elements include diverse lambda-like prophages that package an approximately 65 kb viral genome called Phage WO, which includes a unique eukaryotic association module, or EAM. The EAM encodes unusually large proteins thought to mediate interactions between the bacterium, its virus, and the host eukaryotic cell. We propose that in addition to conventional bacteriophages, *Wolbachia* also encode smaller gene transfer agents (GTAs). From two independent preparations, we recovered an identical 15.6 kb sequence that lacks an EAM. Its small size and gene composition suggest that the element is a GTA, which may participate in the horizontal transfer of random DNA from the *Wolbachia* genome. Although functional proof of GTA activity remains to be obtained, these data provide a useful framework for future analysis of mobile genetic elements encoded by *Wolbachia* genomes.

**Abstract:**

*Wolbachia* are obligate intracellular bacteria that occur in insects and filarial worms. Strains that infect insects have genomes that encode mobile genetic elements, including diverse lambda-like prophages called Phage WO. Phage WO packages an approximately 65 kb viral genome that includes a unique eukaryotic association module, or EAM, that encodes unusually large proteins thought to mediate interactions between the bacterium, its virus, and the eukaryotic host cell. The *Wolbachia* supergroup B strain, *w*Stri from the planthopper *Laodelphax striatellus*, produces phage-like particles that can be recovered from persistently infected mosquito cells by ultracentrifugation. Illumina sequencing, assembly, and manual curation of DNA from two independent preparations converged on an identical 15,638 bp sequence that encoded packaging, assembly, and structural proteins. The absence of an EAM and regulatory genes defined for Phage WO from the wasp, *Nasonia vitripennis,* was consistent with the possibility that the 15,638 bp sequence represents an element related to a gene transfer agent (GTA), characterized by a signature head–tail region encoding structural proteins that package host chromosomal DNA. Future investigation of GTA function will be supported by the improved recovery of physical particles, electron microscopic examination of potential diversity among particles, and rigorous examination of DNA content by methods independent of sequence assembly.

## 1. Introduction

*Wolbachia pipientis* (Rickettsiales; Anaplasmataceae) is an obligate intracellular alpha-proteobacterium that occurs in members of the Ecdysozoa, the group of molting animals that includes arthropods and nematodes. The abundance and diversity of *Wolbachia*’s hosts, horizontal transfer among *Wolbachia* genomes, and transinfection between species to produce novel infections for the reduction in vector-borne diseases have stimulated interest in the genetic manipulation of *Wolbachia*.

Genomes of *Wolbachia* that infect arthropods as reproductive parasites typically include one or more distinct prophage regions, or haplotypes, that vary in overall gene composition, but often share nearly identical copies of individual phage-related genes. In general, these prophages resemble the lysogenic form of bacteriophage lambda [1,2,3,4], with genomes of approximately 65 kb that include a unique eukaryotic association module, or EAM [3,4]. Electron microscopic observation of virus-like particles in *Wolbachia*-infected hosts is consistent with lytic replication of WO prophages and their potential value as transducing agents [1]. Experimental evaluation of phage production from *Wolbachia* genomes is challenging because conditions that induce lytic replication of WO prophages are unknown, and viral particles are recovered at low yields. These limitations likely reflect *Wolbachia*’s obligate intracellular lifestyle and genome streamlining, including the loss of bacterial genes involved in phage induction in free-living microbes [5,6].

Here, we describe the recovery of physical particles produced by supergroup B *Wolbachia* strain *w*Stri, from the planthopper *Laodelphax striatellus*, maintained in the C/*w*Stri1 mosquito cell line [7,8]. Two independent preparations from persistently infected cells converged on an identical 15.6 kb sequence encoding 22 genes, highly similar to the proximal region of WOVitA1 and the corresponding sequence in other well-defined WO phages. Unlike Phage WO, the 15.6 kb sequence, here called WOStri, lacked genes encoding the EAM and regulatory functions; in size and gene composition, WOStri resembled the head–tail gene cluster of a gene transfer agent (GTA) capable of packaging host DNA [9,10,11,12,13,14].

We note that precise linear correlation of DNA sequences recovered from physical particles with a corresponding prophage haplotype awaits progress in the annotation of the *Wolbachia* genomes from which WO phages have been recovered. Moreover, the possibility that *Wolbachia* genomes encode mobile genetic elements other than bacteriophages is supported by bioinformatics-based analyses [9,15,16]. These observations underscore the need for improving yields of virus-like particles produced by *Wolbachia*, and uncovering conditions that enhance their expression. We suggest that systematic implementation of experimental approaches that differentiate between prophages, GTAs, and other virus-like elements will provide new insights into the potential diversity of *Wolbachia*-encoded mobile elements and their genetic capabilities.

## 2. Materials and Methods

### 2.1. Cells and Culture Conditions

C/*w*Stri1 cells were grown in E5 medium as described previously [7,8]. Recovery of approximately 100 ng of phage DNA after ultracentrifugation was guided by proteomic data predicting low phage abundance [17], an estimated harvest of 3 × 10^6^ cells per ml, an assumed abundance of 100 *Wolbachia* and one phage per cell, and a phage genome size of 40 kb.

### 2.2. DNA Extraction and Purification

DNA was extracted from one to three liters of material pooled from 40 to 120 plates with a diameter of 100 mm. Cells were lysed by addition of 0.1 volume of chloroform with vigorous stirring on a magnetic stirrer (30 min at room temperature). Chloroform was removed by low-speed centrifugation, and the supernatant was supplemented with one-fifth volume of 2.5 M NaCl containing 37.5% polyethylene glycol 8000 (PEG) added with stirring at 4 °C. Individual preparations were stored at 4 °C to accumulate sufficient cell culture supernatant. Supernatants were combined, and care was taken to recover a light film of material that adhered to the bottom of glass storage bottles. PEG pellets were recovered after centrifugation in 250 mL bottles in a JA14 rotor (7500× *g* in a J2-21 Beckman centrifuge) and were resuspended in 20 mM Tris-HCl, pH 7.5 containing 10 mM MgCl_2_ and 10% glycerol (approximately 70 mL/L of cell culture supernatant). PEG was extracted from the resuspended material by stirring with an equal volume of chloroform, followed by centrifugation to remove chloroform. The aqueous supernatant was filtered through 5-micron filters, followed by a second filtration through a stacked series of 5-micron, 2.7-micron, and 0.22-micron filters. The filtrate was centrifuged in an SW41 rotor at 110,000× *g* for 16 h at 4 °C. DNA was extracted from pellets by conventional phenol extraction/ethanol precipitation (2016 sample) or with a PureLink (Invitrogen, Carlsbad, CA, USA) genomic DNA extraction kit (2020 sample) following the manufacturer’s instructions. Phage enrichment was verified by a positive PCR band for “orf7” defined for WO phages from *w*Tai and *w*Kue [18], using forward primer 5′-CTGSCTTCAAGKTGCTTTATTGC and reverse primer 5′-TCAAGAGAYCARATAACAGTAGC, coupled with absence of a PCR band corresponding to the single copy *Wolbachia* ribosomal protein gene pair using the S12F/S7R primers described previously [19]. Serial dilution of template estimated phage enrichment at 1000 to 10,000-fold.

### 2.3. Analysis

Samples were submitted to the University of Minnesota Genomics Center for Next-Gen Illumina paired-end sequencing. The 2016 library was prepared using NexteraXT reagent kits, and the 2000 library was created using Rubicon reagent kits. Both projects were sequenced on a MiSeq 300 bp PE stowaway run, and each library generated ~2 M reads, with a mean quality score ≥ Q30. FASTQ data were groomed and trimmed, yielding 302,155 sequences in 2016 and 560,466 sequences in 2020, with an average read quality score of 37 and GC content of 36%. Read length was approximately 300 bp. Assembly was performed using SPAdes (Galaxy version 3.15.3 + galaxy1; [20]). This analysis involved systematic comparison using increasing coverage criteria until the assembled reads were represented by a maximum of three contigs from the second independent preparation: 10.29_1, coverage 3425; 10.29_2, coverage 1043; and 11.1_1, coverage 4042, obtained from 10.29_1 and 10.29_2 by raising coverage stringency. At this step, we excluded the lower coverage 10.29_2 contig based on the presence of an IS110 transposase and a unique HP in reverse orientation relative to other genes in the three contigs. The 2020 data were then reanalyzed against the single 2016 contig that emerged from a high coverage analysis. An identical 15,638 nt core was represented by both data sets. The 2020 data varied at the flanking 5′ end of this core sequence, while the 2016 data varied at the flanking 3′ end. To facilitate description of the results here, we call the 15,638 nt core sequence “WOStri”, but acknowledge that in overall size and gene composition, it bears stronger resemblance to a GTA than to a conventional WO Phage [3,4].

Data were compared and manipulated using Geneious Prime (version 2022.0.2) [21,22] and Blast programs on NCBI (National Center for Biotechnology Information) [23,24], with a primary focus on the well-characterized prophage WOVitA1 (66,810 bp, HQ906662.1), alternatively known as Phage WO (KX522565.1). Queries were generated by Geneious Prime from translation products encoded by WOStri orfs. We evaluated alignments from the following complete, or nearly complete genomes: *Wolbachia* supergroup A strains *w*Mel (NC_002978.6; taxid 163164), *w*Ri (NC_012416.1; taxid 66084), *w*Ha (NC_021089.1; taxid 1236909), and *w*Au (NZ_LK055284.1; taxid 225364); supergroup B strains *w*No (NC_021084.1; taxid 1236908), *w*Pip (NC_010981.1; taxid 570417), and *w*StriCN (NZ_ MUIX01000001.1 and NZ_MUIX01000002.1; taxid 368602); and supergroup E strain *w*Fol (NZ_CP015510.2; taxid 169402). Note that the *w*StriCN genome [25] is represented by two contigs, NZ_MUIX01000001.1 and NZ_MUIX01000002.1; these are abbreviated “MUIX_01.1” and “MUIX_02.1” in the text below. The gene composition of the WOStri core has been deposited in Genbank (accession number: BankIt2634826 BSeq#1 OP690541, to be released in April 2023).

## 3. Results and Discussion

### 3.1. WOStri Resembles the Proximal Region of Phage WO

**Figure 1:**
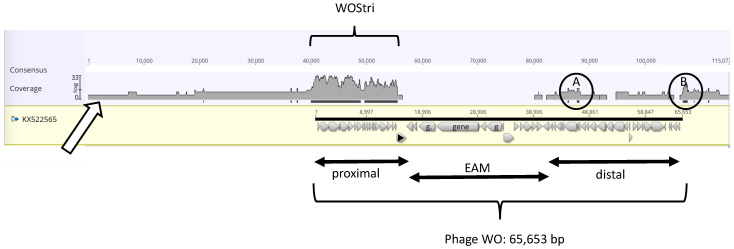
Combined SPAdes 2016 and 2020 reads mapped to Phage WO (KX522565). Panel A. Geneious Prime “Map to reference” mapped 523 of 3499 contigs generated without a coverage cutoff to Phage WO (KX522565). Introduction of gaps to maximize the alignment lengthens the consensus from 65,653 nt to 115,077 nt. The open arrow indicates the coverage profile (Log scale, shaded in gray) of mapped reads, and black bars beneath the profile indicate coverage with 2 or more reads. Annotation of Phage WO is indicated by gray arrows beneath the black bar, with proximal, EAM, and distal regions indicated below the alignment. Note that short areas of two-fold coverage (circled) occur within (A) and just downstream (B) of the KX522565 sequence.

We compared DNA recovered from two independent preparations of physical particles from *w*Stri, a supergroup B *Wolbachia* strain from the planthopper *Laodelphax striatellus* (Hemiptera, Delphacidae) maintained as a persistent infection in *Aedes albopictus* C/*w*Stri1 mosquito cells [7,8]. Because an earlier proteomics investigation [17] showed evidence for the expression of phage peptides that map in the Undecim cluster [4], we anticipated recovery of a lambda-like genome resembling Phage WO (KX522565.1: 65,653 bp), encoded by prophage WOVitA1 (HQ906662.1: 66,810 bp) in *Wolbachia* that infect the wasp, *Nasonia vitripennis* [3,4]. Illumina data were groomed, trimmed, and assembled using SPAdes without a coverage cutoff, and contigs generated from the combined data sets were aligned to Phage WO using Geneious Prime (Figure 1).

The reads predominantly mapped to a 17 kb proximal region of Phage WO, bracketed as WOStri. The 65,653 bp Phage WO genome, bracketed at the bottom of Figure 1, is represented by a solid black bar above a series of annotated genes denoted by gray arrowheads. The proximal, or 5′ region, extends from hypothetical protein (HP) gwv_1137 to recombinase (gvw_1156). Note that the gray-shaded read profile is shown on a log scale with a Geneious Prime coverage of “30” in the WOStri region, and that high coverage does not include the Phage WO recombinase (marked with a black triangle), the eukaryotic association module (EAM), or the distal portion of Phage WO. Two minor exceptions are represented by black bars identifying circled areas A and B, which had a Geneious Prime coverage of “2”.

Region A maps in the vicinity of gvw_1110 to gvw_1113 in Phage WO, encoding the P2 ortholog gpU, the tape measure protein (described in further detail in Section 3.5), and tail chaperone proteins gpGT and gpG. Circled region B maps downstream of the last annotated gene (gwv_1134). Within this 457 nt region are several short orfs measuring less than 200 nt. The sequence is represented by a series of 344 repeats, each measuring 5 bp and occurring as two to six copies. Blastn shows that a similar sequence is common among *Wolbachia* genomes but has not been identified in *w*Stri. In *w*Pip, the orthologous sequence occurs as four copies of a putative phage-related protein in the RNAse H-like superfamily. These isolated, low-abundance regions of phage-like genes fell out of the assembly as coverage stringency increased.

### 3.2. WOStri Gene Order and Sequence Are Conserved in Other WO Phages

**Figure 2:**
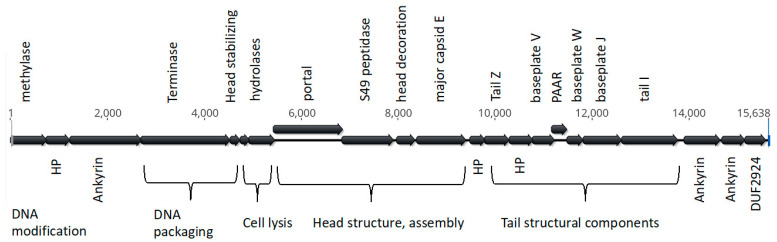
Structure of WOStri. Open reading frames were produced in Geneious Prime. Genes clearly identifiable as phage-related are listed above, and unknown genes are labeled below the alignment.

By systematically increasing the coverage cutoff in SPAdes from 0 to 3000, we found that both data sets converged on an identical 15,638 nt sequence encoding 22 genes oriented in the same direction (Figure 2).

**Figure 3:**
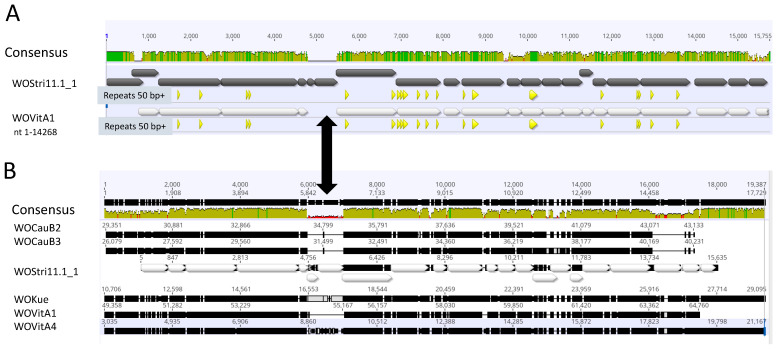
Overall sequence comparisons. (**A**) Pairwise alignment of WOStri 11.1_1 with residues 1–14,268 of KX522565. Green/yellow-green and red shading indicate Geneious designations of high, medium and low sequence identity. Gray (WOStri) and white (WOVitA1) horizontal arrows represent orfs and their direction of transcription. Triangles below the orfs indicate exact repeats of 50 nt or greater common to WOStri and the proximal region of KX522565. The double black arrow connecting Panels A and B indicates the variable region separating terminase/gpw and portal genes in *Wolbachia* prophages. (**B**) Sequence alignment of WOCauB2 (AB478515, bases 29,351–43,133); WOCauB3 (AB478516, bases 26,079–40,231); WOStri and WOKue (AB036666, bases 10,706–29,095); WOVitA1 (HQ906662, reversed, bases 49,358–64,760); and WOVitA4 (HQ906664; reversed, bases 3035–21,167). The region common to all six genomes was extracted and aligned in Geneious Prime.

We note that the methylase at the extreme 5′ end of WOStri (Figure 2) has an ortholog downstream of *attL* at the extreme 5′ end of the integrated WOVitA1 prophage that is not annotated in KX522565. N-terminal methylase variants with legitimate bacterial start codons encoded proteins ranging from 125 to 410 amino acids. The N-terminal domain of the longer orthologs included a ParB_N_Srx domain of about 100 residues, which may encode nuclease activity. With the exception of hydrolase/s (orfs6/7), orfs downstream of the methylase closely resembled a 14,262 region of Phage WO that includes a series of shared repeats measuring 50 bp or more (Figure 3A).

The 15,638 nt region was conserved among other well-annotated WO phages, including WOVitA1, WOCauB2, WOCauB3, WOVitA1, as well as the smaller WOKue and WOVitA4 (Figure 3B). Each of the WOStri genes had one or more orthologs in *w*Stri and, with a few individual exceptions, in other well-characterized *Wolbachia* genomes, as summarized in Table 1.

Assemblies of the host *Wolbachia* genomes from which phages have been recovered are incomplete, and to date, none of the phage structures resulting from conventional molecular approaches and/or assembly of short reads has been correlated with a contiguous suite of genes in a host genome. In aggregate, *w*Stri encodes nine putative phage regions, representing more than 10% of the genome [25]. Although all of these prophage regions include genes classified as “other than prophages and transposons”, we note that six of the nine putative prophages have estimated sizes ranging from 16.7 to 20.6 kb, comparable in size to WOStri and WOVitA4. The size of WOStri, absence of regulatory genes, and identities of structural genes involved in DNA packaging suggested that WOStri may be a gene transfer agent, similar to that in *Rhodobacter capsulatus*, a marine alpha-proteobacterium which is well studied because a GTA overproducer strain is available [10].

### 3.3. The WOStri Hydrolase Region Contains Evidence for a +1 Programmed Ribosomal Frameshift

Alignment with other WO Phages reveals a single variable region (Figure 3) between terminase/gpw (orfs 4/5) and portal (orf 8) genes, which may be absent, may encode a GH_25 hydrolase/muramidase, an alternative PD-(DE)XK nuclease, or a hypothetical protein and/or a RelE/ParE toxin-antitoxin module, which have been described elsewhere [26]. WOStri most closely resembles WOVitA4 (Figure 3B), which also encodes a GH_25 hydrolase/muramidase. Phage WO, WOCauB2, and WOCauB3, best known for their EAM regions [3,4], lack an intervening sequence, while WOKue encodes a PD-(D/E)XK nuclease.

**Figure 4:**
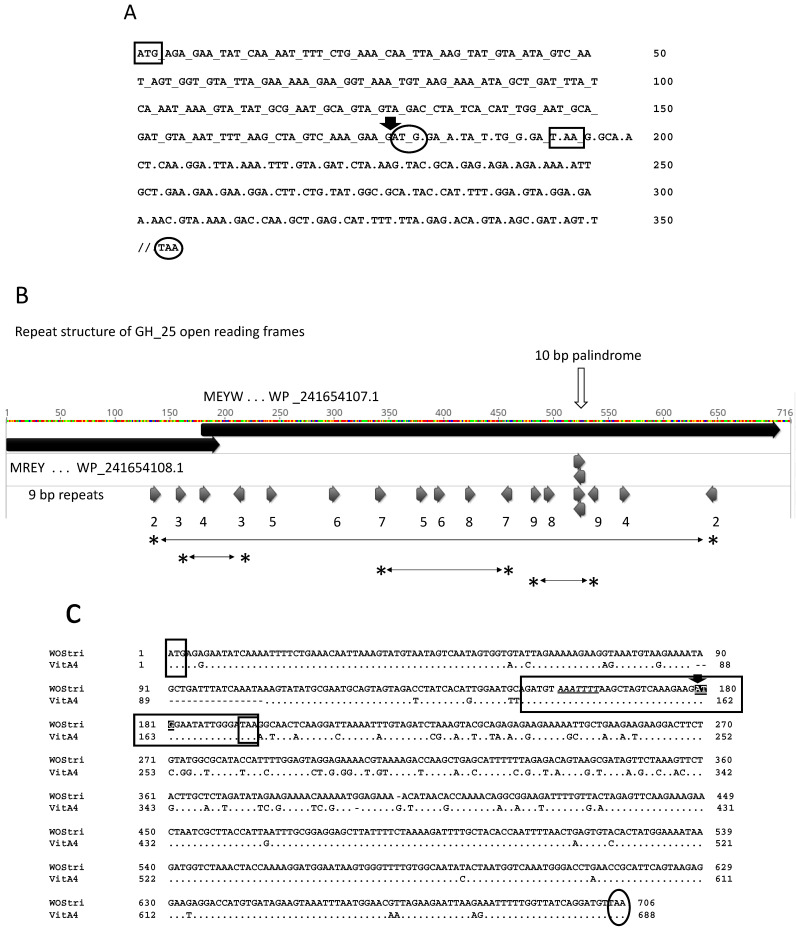
Sequence support for a programmed + 1 ribosomal frameshift in hydrolase-encoding orfs. Panel (**A**) shows the sequence of the hydrolase Orf6 and Orf7 in the region of the potential frameshift. Rectangular boxes show initiation and termination codons of Orf6, and oval boxes show the start and stop codons of Orf7. The downward pointing arrow indicates the potential + 1 frameshift. Codons in the Orf6 reading frame are separated by underscores, and codons in the Orf7 reading frame are separated by periods. The 3′-end of Orf7 is not shown. Panel (**B**) shows a translation of GH_25 Orf6/Orf7 using Geneious Prime. Colors are the Geneious designations for residues and are not relevant here. Large solid black bars indicate the two translation products: N-terminal MREY… and MEYW…, with WP_ accessions. Short arrows below indicate positions of 9 and 10 bp repeats in the Orf6/Orf7 region, suggesting possible secondary structure in the mRNA. Horizontal lines flanked by asterisks show sequence flanked by repeat pairs 2, 3, 7, and 9, oriented in opposite directions. The white vertical arrow indicates a 10 nt palindrome. Panel (**C**) shows alignment of hydrolase reading frames in WOStri and WOVitA4. Dots indicate identities. Small rectangular boxes with only three nucleotides represent ATG start and TAA stop codons in Orf6. The region of exact identity immediately surrounding the out-of-frame Orf7 ATG (downward pointing arrow and white text on a black background) is shown in the long horizontal rectangle that terminates with the Orf6 TAA stop codon. Within the box, a slippery sequence *AAATTTT* upstream of the start codon is shown in italics and underlined. The oval represents the Orf7 stop codon.

The evolution of bacterial muramidases/hydrolases has been described earlier [36], and we suggest that in the context of a GTA, the hydrolase may contribute to host cell lysis. The *w*Stri ortholog WP_241654108.1 (orf6, 64 amino acids), has a single putative hydrolase domain (residues 42–62); in WP_241654107.1 (orf7, 175 amino acids), the hydrolase domain extends from residues 5 to 160. We note that a +1 ribosomal frameshift (Figure 4A) potentially produces a translation fusion protein from orfs6/7.

Although signals in the mRNA that might trigger a +1 ribosomal frameshift are not well understood, the sequence includes a 10 bp palindrome and several 9 bp repeats (Figure 4B), some of which are in reverse orientation and potentially generate secondary structure in mRNA transcripts [37]. A nucleotide alignment with the similar hydrolase coding region in WOVitA4 includes a region of identity spanning the position of the potential frameshift, with the potential XXXYYYN “slippery sequence” best associated with −1 ribosomal frameshifts, shown in underlined italics upstream of the ATG start codon (Figure 4C). Bacterial genes with orfs disrupted by programmed ribosomal frameshifting and programmed transcriptional realignment are apparently common, but difficult to identify computationally [38].

By way of comparison, the RcGTA gene responsible for bacterial lysis has not been unequivocally identified [39]. However, experimental evidence suggests that an unknown protein with a glycohydrolase domain encoded by VSH-1, a GTA in the anaerobic spirochete *Brachyspira hyodysenteriae*, hydrolyzes peptidoglycan [40]. We speculate that the muramidase/hydrolase motifs encoded between terminase/gpW and portal genes may play a role in WOStri release, and that variation at this locus may have physiological significance.

### 3.4. Evidence for Low-Level Packaging of wStri Genomic DNA Encoding EAM-like Proteins

We suggest that the 15,638 bp sequence we call WOStri represents an equivalent of the minority of RcGTA particles in which the head–tail encoding genome is packaged [11,14]. As a GTA, WOStri would have also packaged random pieces of the *w*Stri genome, albeit at low frequency for any particular DNA fragment. Although not conclusive, examination of the low abundance reads is informative in the context of future experimental investigation. In general, these data are consistent with low coverage of *w*Stri DNA. Moreover, early steps in the assembly generated contigs that encoded long orfs reminiscent of EAM proteins. These observations merit more rigorous future investigation.

**Figure 5:**
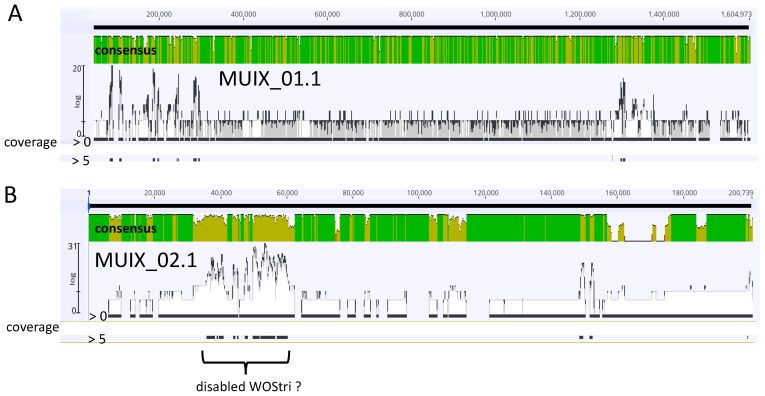
Mapping of combined SPAdes contigs without coverage cutoff to MUIX_01.1 (Panel (**A**)) and MUIX_02.1 (Panel (**B**)) contigs representing an incomplete *w*Stri genome. Green/yellow-green and red shading in consensus panels indicate Geneious designations of high, medium and low sequence identity. Of 3499 total reads, 1193 mapped to MUIX_01.1, and 515 mapped to MUIX_02.1. Profiles show consensus identities generated with Geneious Prime, with highlighted coverages set to >0 (highest possible representation of matches between contigs and *w*Stri DNA) and to >5, represented by black bars below the top and lower consensus bars, respectively. Distribution of coverage bars emphasizes potential coverage of most of the *w*Stri genome with the complete set of SPAdes contigs, and loss of matches as Geneious coverage criteria increase.

Although we cannot rule out adventitious contamination that would be eliminated by rigorous DNAse treatment of purified particles, at the lowest coverage, the complete set of reads showed wide distribution across the two contigs that represent the *w*Stri genome (Figure 5).

These reads are represented by the solid black bar immediately below each panel, labeled coverage “>0.” When coverage was raised to “5”, most of these reads were eliminated, while a region in MUIX_02.1 remained, possibly representing a rearranged WOStri (Figure 5B). The region encodes a discontinuous suite of the WOStri genes reported here, all oriented in the same direction, but including two copies of an IS110 family transposase (WP_063630491.1) and a duplication of baseplate assembly genes V and W. Moreover, the portal gene directly follows terminase/gpW genes, and a recombinase occurs immediately downstream of a DUF2924 domain-containing protein.

In addition to the possibility that *w*Stri genomic DNA is packaged, long (>5000 nt), low abundance contigs sometimes encoded single orfs reminiscent of genes in the EAM or distal regions of Phage WO. These long, low-frequency contigs, presumably representing intermediates produced by the assembly algorithm, encoded three classes of proteins: ankyrin motif proteins, ATPases associated with diverse cellular activities (AAA-ATPases), and phage tape measure proteins (Table 2).

These proteins have internal repetitive domains and participate in protein–protein interactions that involve the assembly of macromolecular structures. We did not find orfs that encoded proteins from host mosquito DNA, which would be expected if extraneous DNA contaminated physical particles recovered by ultracentrifugation.

### 3.5. Evaluation of EAM-like Proteins

Ankyrin repeat and latrotoxin-associated proteins included an exceptionally large (11,826 nt) orf encoding a 3941 residue protein reminiscent of the 2474 residue ankyrin/tetratricopeptide repeat protein in WOVitA1. This protein was identical to the ankyrin repeat protein WP_063631194.1 from the Korean strain of *w*Stri (NZ_LRUH01000086.1), but is not encoded in the more complete *w*Stri MUIX_01.1 and _02.1 assemblies, nor is it annotated among the selected *Wolbachia* strains examined here. However, orthologs with lower coverage/identity scores were common, including *w*Stri WP_143688882.1, with 16 matches over 4357 residues, and WP_143688758.1, with 12 matches over 3082 residues.

Three AAA-ATPases are annotated in the distal portion of WOVitA1 (gwv_1124, gwv_1125, and gwv_1128). These proteins are characterized by a conserved 230 amino acid ATPase module which acts as a motor to drive macromolecular rearrangements in diverse cellular functions [41,42].

Finally, the tape measure protein (TMP), often recognized by the distribution of aromatic amino acids, is common in prophage genomes, typically measures about 1000 residues, and, like ankyrin proteins, contains partially repeated regions [43,44]. During infection, TMP controls phage tail length and facilitates DNA transit to the bacterial cytoplasm [45]. Uniquely among the large proteins considered here, tape measure proteins are encoded by eight genes distributed among the two *w*Stri MUIX contigs, one of which (WP_077188535.1) shared complete identity with the WOVitA1 query. In contrast, however, *w*Mel, *w*Ri, *w*Ha, *w*Au, and *w*Pip genomes encoded only a single ortholog, while four were present in *w*Fol.

The 2020 data set encoded a total of 27 orfs exceeding 2000 nt in length, again with no evidence of contaminating mosquito sequences. Of these, six were duplicates of ankyrin, AAA-ATPase, and TMP genes from the 2016 data set (indicated in bold font in Table 2). The remaining orfs were encoded by genes present as one to two copies in the *w*Stri genome, and included non-phage proteins such as RNA polymerase, RecB, and a translational initiation factor. Recovery of contigs encoding EAM-like proteins only in the early stages of assembly may derive from the overrepresentation of some, but not all, regions of the *w*Stri genome. For example, a GTA from flea-borne *Bartonella grahamii* is associated with packaged DNA that includes products generated by run-off replication of a chromosomal “high plasticity zone” thought to facilitate diversification of the host microbe [46,47].

## 4. Conclusions

We present an analysis that supports the possibility that GTA-like elements may be encoded in *Wolbachia* genomes and provide insights that serve as a baseline for further investigation of this possibility. In this context, it is important to note that GTA-like elements that do not transfer DNA, such as the defective phage PBSX from *Bacillus subtilis,* have been described [10], and that an understanding of GTA expression and function continues to evolve, as has recently been demonstrated for *Bartonella* GTA [48]. In addition, subtle differences in the structure of physical particles can correlate with packaging capability [14]. The sequence assembly of DNA recovered from particles produced by *w*Stri in cultured cells was unexpectedly complex. Generation of a single consensus sequence required multiple iterations with progressively increased stringency, in which the longest contigs, some with orfs resembling genes encoded by the EAM in Phage WO, were not retained in the final assembly. Although we cannot eliminate an assembly artifact and/or potential contamination of phage-like particles with *w*Stri genomic DNA, recovery of an identical 15.6 bp WOStri core sequence from two independent investigations, coupled with its strong similarity to the proximal portion of WOVitA1 and other WO phages, merits further analysis using more direct approaches that complement computational assembly, including direct sequencing of *w*Stri chromosomal fragments from physical particles, as has been performed with RcGTA [10,11,12,13,14]. In addition, high-quality transmission electron microscopy of *Wolbachia* preparations will be important to evaluate the diversity of phage-like particles [14]. Finally, more rigorous purification of packaged DNA by cesium chloride gradient centrifugation, ideally under conditions that separate particles based on density and/or structural differences, will be desirable. These endeavors will require scaled-up procedures for particle recovery and, ideally, the identification of conditions that induce the expression of phages and/or GTAs in C/*w*Stri-infected cells. Some GTAs may be inducible by nutrient depletion or quorum sensing [15], while DNA damage can elicit a lytic cycle in some bacteriophages. In pilot experiments, we have tested exposure to ultraviolet light, mitomycin C, and oxidizing agents, thus far without successful induction. Regardless of the precise nature of potential agents of gene transfer in *Wolbachia*, further exploration of their identities will contribute to the eventual genetic manipulation of this widespread intracellular bacterium.

## Figures and Tables

**Table 1:** WOStri orthologs in *w*Stri and other *Wolbachia* strains. Note that the prefix WP_ is omitted from accession numbers. The query was the translation product from the WOStri orf, with the exception of the methylase (orf1), where the query was the WOVitA1 ortholog.

WOStri	Accessions WP_										
(orf)	*w*Stri	Vit	MUIX_	wMel_A	wRi_A	wHa_A	wAu_A	wPip_B	wNo_B	wFol_E	WOStri Comments
orf1, DNA methylase	143688966.1		_02.1	010962700.1	006280179.1	015589002.1	CDR79043.1	007302866.1	015588411.1	110410402.1	length varies; N-terminal ParB_N_Srx doman; WOVitA1 ortholog gwv_1137 is query
	143688770.1		_01.1	010962471.1		015588907.1	CDR78683.1	007302943.1		110410376.1	
	143688913.1		_01.1				CDR78684.1			110410091.1	
	143688988.1		_02.1								
orf2, HP	143688769.1		_01.1	010962701.1	006280180.1	010962472.1	010962472.1	012481828.1	015588412.1	110410601.1	
	143688965.1		_02.1	022626311.1	007549853.1	015589000.1	CDR78685.1	007302942.1	015587745.1	110410401.1	
	143688914.1		_01.1	010962472.1						110410092.1	
	077188538.1	VitA1	_01.1, _02.1	010082154.1							
orf3, Ank repeat	143688768.1		_01.1	010962702.1	010082188.1	015588999.1	010082188.1	012481772.1	015587744.1	110410093.1	
	143688726.1		_01.1						015588413.1	110410375.1	
	077188539.1	VitA1	_01.1							110410603.1	
	143688935.1		_01.1								
	162848928.1		_01.1								
orf4, Terminase	143688923.1		_01.1	010962703.1	012673198.1	015588998.1	015588914.1	012481771.1	015588414.1	110410400.1	[26]
	143688915.1		_01.1	010962473.1	041582652.1	015588914.1	CDR78687.1		015587743.1	110410094.1	
	077188540.1		_01.1, _02.1		007548331.1					110410374.1	
	241654230.1		_02.1								
orf5, gpW	143688767.1		_01.1	010962704.1	010962704.1	010082193.1	010082193.1	007302777.1	015588415.1	110410373.1	[26]
	143688727.1		_01.1	010082193.1	007549008.1		CDR78688.1	007301848.1	015587742.1	110410095.1	
	143688830.1		_01.1		007548330.1			007302937.1		110410399.1	
	077188541.1	VitA	_01.1, _02.1					044475425.1			
orf 6, Hydrolase	241654108.1		_01.1		006280957.1				015588109.1		[26]; Figure 3 and Figure 4
	241654159.1		_01.1		238514242.1						
	241654099.1		_01.1								
orf7, Hydrolase	241654107.1				006280957.1				015588109.1		
	241654099.1				238514242.1						
orf8, Portal	143688977.1		_02.1		012673196.1	015588997.1	CDR78691.1	007302936.1	015588417.1	110410397.1	[26]
	096617421.1		_02.1		007549005.1		CDR79035.1	007301845.1		110410096.1	
	143688728.1		_01.1		039963649.1		CDR79034.1	012481828.1		110410371.1	
	143688827.1		_01.1								
	143688928.1		_01.1								
orf 9, S49 peptidase	143688824.1		_01.1	006280203.1	012673244.1	015588996.1	006280203.1	012481770.1	015587741.1	110410396.1	
					006280203.1	015588915.1	CDR79033.1	012482035.1		110410097.1	
								012481827.1		110410370.1	
orf 10, Head decoration	143688823.1		_01.1	010962475.1	007548326.1	015588917.1	CDR79031.1	012482034.1	015588497.1	110410369.1	
	010405448.1		_01.1	010082142.1	007549003.1	015588995.1	010082142.1	007302934.1	015587740.1	110410395.1	
	143688729.1		_01.1		010082142.1			007302870.1		110410098.1	
orf 11, Major capsid E	010405450.1	VitA,B	_01.1	010962707.1	007548999.1	015588918.1	006280204.1	012481769.1	015587739.1	110410368.1	possible role in abortive infection [27,28,29,30]
	143688822.1		_01.1	010962476.1	006280204.1		CDR79030.1	012482033.1		110410394.1	
					007548325.1			012481826.1		110410579.1	
								012481801.1			
orf 12, HP	143688821.1		_01.1	007550857.1	007550857.1	010082171.1	007550857.1	007301839.1	015588264.1	110410393.1	
	143688715.1		_01.1	010082171.1	012673243.1		010082171.1	012481768.1	015587738.1	110410099.1	
	143688795.1		_01.1		007548323.1			CAQ54530.1		110410367.1	
	143688961.1		_02.1					007302873.1			
								012481800.1			
orf13, Tail Z	143688820.1		_01.1	038228285.1	006280676.1	010962479.1	010962479.1	012481823.1	015588265.1	110410392.1	Head–tail joining [31,32]
	143688765.1		_01.1	010962479.1	006280854.1		006280676.1	007302874.1	015587737.1	110410100.1	
	143688714.1		_01.1					007301838.1		110410366.1	
	143688794.1		_01.1					007302931.1			
	143688960.1		_02.1								
	010405454.1	VitA1	_01.1								
ore 14, HP	143688818.1		_01.1	010962480.1	010962732.1	015588919.1	010962732.1	007301837.1	015588266.1	110410101.1	
	143688713.1		_01.1	010962732.1	006280893.1		CDR79025.1	007302875.1	041581447.1	110410365.1	
	010405456.1		_01.1					012481799.1		110410391.1	
	143688793.1		_01.1, _02.1								
	143688964.1		_02.1								
orf15, baseplate V	015588267.1		_01.1	010962731.1	012673195.1	015588920.1	012673195.1	007302876.1	015588267.1	110410390.1	tailocins [33,34,35]
	143688792.1		_01.1, _02.1		007548312.1		CDR79024.1	012481798.1	015587735.1	110410575.1	
	096641463.1		_01.1		007548997.1			012482032.1		110410102.1	
	143688963.1		_02.1							110410364.1	
orf 16, PAAR	143688817.1		_01.1	010082105.1	010082105.1	015588921.1	010082105.1	012481797.1	015588268.1	110410103.1	[10,33,34,35]
	143688962.1		_02.1	010082109.1	007548996.1		010082109.1	007302877.1	015587734.1	110410389.1	
	063630987.1		_01.1, _02.1		049749693.1			012482031.1		110410000.1	
	010405460.1	VitA1	_01.1								
	143688712.1		_01.1								
orf 17, Baseplate W	143688711.1		_01.1	007552425.1	007548437.1	015588922.1	007552425.1	007302878.1	015588269.1	110410104.1	
	010405462.1	VitA1	_01.1	015588922.1	007552425.1		015588922.1	012481796.1	015588498.1	110409999.1	
					012673242.1						
orf 18, Baseplate J	143688710.1		_01.1	010962482.1	012673402.1	015588994.1	010962482.1	012481795.1	015588270.1	110409998.1	
	143688959.1		_02.1	010962729.1	012673194.1	015588923.1	012673194.1	007301818.1	015587733.1	110410388.1	
	143688789.1		_01.1	012673194.1	012673241.1			012481767.1	015588499.1	110410105.1	
orf 19, Tail protein I	143688764.1		_01.1	015588924.1	012673401.1	015588993.1	CDR78703.1	007302879.1	015588271.1	110409997.1	
	143688709.1		_01.1	010962728.1	006280238.1	015588924.1	CDR79020.1		015587732.1	AWW50841.1	
	143688958.1		_02.1		012673240.1				015588500.1		
	077188546.1	VitA1	_01.1								
	143688788.1		_01.1								
ore 20, HP	143688763.1		_01.1	041580639.1	006280237.1	015588992.1	CDR78705.1	007302881.1	015587731.1		
	143688865.1		_01.1					044475433.1	015588272.1		
	143688708.1		_01.1								
	177429427.1		_01.1								
	177429437.1		_02.1								
orf 21, HP	019236494.1		_01.1	010962726.1	007549613.1	015588273.1	CDR78707.1	007302927.1	015587730.1		
	143688707.1		_01.1					007302882.1	015588273.1		
	143688864.1		_01.1								
	143688957.1		_02.1								
orf 22, DUF 2924	143688762.1		_01.1	010962725.1	044471239.1		CDR78708.1	007302926.1	015587729.1	110409343.1	
	143688785.1		_01.1	010962486.1	015588926.1		CDR79016.1	007302883.1		110409994.1	
	143688706.1		_01.1				015588926.1			110409273.1	
										110410108.1	

**Table 2:** Analysis of low coverage contigs exceeding 5000 nt in length for large open reading frames (exceeding 2000 nt) potentially encoding EAM-like proteins. Entries in bold are common to both data sets. The final column indicates that ankyrin proteins may be represented by many additional putative orthologs at less than 50% coverage or identity.

								Copies	Copies	Coverage/Identity
Contig Number	Length	Coverage	Orf Length	*w*Stri Protein Accession	Coverage (%)	Identity (%)	Function	MUIX	Non-MUIX	<50%/50%
**2016 data**										
node 1	15,817	27.7	2253	WP_143688936.1 01.1	92	99	AAA-ATPase	2		
node 2	14,479	59.8	11,826	**WP_063631194.1 LRUH**	100	100	Ankyrin		1	
node 3	13,399	17.5	5772	**WP_206663913.1 01.1**	98	100	Ankyrin	1		
node 4	11,891	19.2	2151	**WP_063630525.1 LRUH**	98	100	AAA-ATPase		1	
node 5	10,094	10.9	2307	WP_143688736.1 01.1	99	99	tape measure	8		
node 6	9405	9.9	3429	**WP_143688778.1 01.1**	98	81	Ankyrin	2		many <50%
node 7	9118	3.9	5730	**WP_143688882.1 01.1**	100	100	Ankyrin	2		
node 8	7970	37.5	2439	**WP_143688947.1 02.1**	99	100	tape measure	8		
**2020 data**										
node 1	36,552	184	2253	WP_063630523.1 01.1	92	100	AAA-ATPase	2		
			2316	WP_143688796.1 01.1	99	100	tape measure	8		
			2601	WP_063630621.1 02.1	99	100	ABC transporter ATP-binding protein/permease	1		many <50%
node 2	30,125	397	2439	**WP_143688947.1 02.1**	99	100	tape measure	8		
			11826	**WP_063631194.1 LRUH**	100	100	Ankyrin		1	
node 3	23,532	**7**	2940	WP_063630441.1 01.1	97	100	DUF 3971	1		
node 4	19,389	153	9243	WP_143688758.1 01.1	100	100	Ankyrin	2		many <50%
node 5	18,215	40.8	2262	WP_063631186.1 01.1	98	100	HP	1		
			3696	WP_177429433.1 01.1	98	100	Collagen-like protein	2		
			5730	**WP_143688882.1 01.1**	100	100	Ankyrin	2		
node 6	17,355	5.8	2712	WP_172793444.1 02.1	99	100	RecB	1		
			3090	WP_063630611.1 02.1	99	100	AIR synthase	1		
node 7	15,996	4.5	8520	WP_084240976.1 01.1	100	100	ββ’ RNA Polymerase	1		
node 8	14,963	148	4470	WP_063631092.1 02.1	99	100	Ankyrin	1		many <50%
node 9	14,237	10.5	2553	WP_063630489.1 01.1	99	100	ATP-dependent chaperone	2		
			4671	WP_063630487.1 01.1	99	100	NAD-glutamate dehydrogenase	1		
node 12	12,174	6.9	2322	WP_063631238.1 01.1	99	100	Translational initiation factor 2	1		
node 13	11,829	155	4245	**WP_143688778.1 01.1**	98	80	Ankyrin	2		many <50%
node 15	10,901	4	2121	WP_143688815.1 01.1	90	95	DNAJ/Ankyrin	1		
node 16	10,612	251	2151	**WP_063630525.1 LRUH**	98	100	AAA-ATPase		1	
node 17	10,406	5.4	2814	WP_063631210.1 01.1	99	100	EXI ABC subunit UvrA	1		
node 18	9228	18.9	3087	WP_143688902.1 01.1	99	100	Ankyrin	2		many <50%
node 20	8085	65.2	7416	**WP_143688882.1 01.1**	99	100	Ankyrin	2		
node 24	7238	3.6	3462	WP_241654150.1 01.1	91	100	DEAD/DEAH box helicase	1		
node 26	6544	16.4	4368	WP_143688901.1 01.1	99	100	Ankyrin	1		
node 28	6264	274	5772	**WP_206663913.1 01.1**	98	100	Ankyrin	1		many <50%
node 34	5542	36	2175	WP_082246120.1 02.1	97	100	AAA-ATPase	1		

## Data Availability

The gene composition of the WOStri core has been deposited in Genbank (accession number: BankIt2634826 BSeq#1 OP690541, to be released in April 2023).
